# *Toxoplasma gondii* sexual cross in a single naturally infected feline host: Generation of highly mouse-virulent and avirulent clones, genotypically different from clonal types I, II and III

**DOI:** 10.1186/1297-9716-43-39

**Published:** 2012-04-30

**Authors:** Daland C Herrmann, Andrea Bärwald, Aline Maksimov, Nikola Pantchev, Majda G Vrhovec, Franz J Conraths, Gereon Schares

**Affiliations:** 1 Friedrich-Loeffler-Institut, Federal Research Institute for Animal Health, Institute of Epidemiology, Seestr. 55, 16868, Wusterhausen, Germany; 2 VetMed Labor GmbH, Division of IDEXX Laboratories, Mörikestr. 28/3, 71636, Ludwigsburg, Germany

**Keywords:** Toxoplasma gondii, Genotype, PCR-RFLP, Cats, Virulence

## Abstract

Tachyzoite clones obtained from a single *Toxoplasma gondii* oocyst field sample were genotyped and characterized regarding mouse virulence. PCR-RFLP genotyping of tachyzoites initially isolated from interferon-γ-knockout (GKO) mice, BALB/c mice and VERO cell culture using the nine independent, unlinked genetic markers nSAG2, SAG3, BTUB, GRA6, c22-8, c29-2, L358, PK1 and Apico revealed mixed *T. gondii* infections showing combinations of type II and type III alleles at different loci. Forty-five individual clones were obtained from all mixed *T. gondii* tachyzoite cell cultures by limiting dilution. Sixteen *T. gondii* clones showed type III alleles at all loci and 29 clones displayed a combination of type II and type III alleles at different loci. Five clone groups were identified in total, four of which include *T. gondii* clones that showed a non-canonical allele pattern and have never been described in natural infections before. All tested clones, except two, were highly virulent in BALB/c mice. The isolation of different non-canonical *T. gondii* clones originating from an oocyst sample of a single naturally infected cat demonstrate that sexual recombination as well as re-assortment of chromosomes via a sexual cross of *T. gondii* occur under natural conditions and result in the emergence of clones with increased virulence in mice.

## Introduction

*Toxoplasma gondii* is an obligate intracellular protozoan parasite infecting all warm blooded mammals and birds worldwide [1]. Upon infection, felids (the definitive hosts) can excrete the environmentally resistant infectious oocyst stage. Intermediate hosts, such as birds, rodents, ruminants and humans may become infected post-natally by either ingesting sporulated oocysts or raw or undercooked meat containing tissue cysts. Drinking water contaminated with *T. gondii* oocysts was the cause of major outbreaks of acute toxoplasmosis [2,3].

Humans may remain infected life-long and often remain asymptomatic unless immunosuppression occurs [4,5]. Only a minority of post-natally infected individuals who contract a *T. gondii* infection may develop symptoms; severe or fatal clinical courses are rare [6]. Congenital toxoplasmosis can occur when a woman is infected with *T. gondii* during pregnancy. Infections passed to the foetus may lead to abortion, stillbirth or a range of manifestations in the foetus or infant which include hydrocephalus, cerebral calcifications and retinochoroiditis [7].

In North America and Europe, three *T. gondii* clonal lineages dominate (types I, II and III) as evident from PCR-RFLP and microsatellite typing [8,9]. Recently, a fourth clonal type was characterised in North America [10]. There is evidence suggesting that *T. gondii* clonal lineages are unevenly distributed in Europe, with non-type II being more prevalent in Portugal and Spain than in Germany [11-15].

While infection with a single parasite of type I is always lethal in outbred mice, more than 10^3^ parasites of types II and III were needed to cause the same effect [16].

Certain alleles of the genes ROP18 (chromosome [Chr.] VIIa) and ROP16 (Chr.VIIb) possess virulence-potentiating properties [17,18]. ROP18 was shown to disarm macrophage clearance by phosphorylating immunity-related GTPases thereby facilitating survival of *T. gondii* in naïve IFN-γ-activated monocytes [19,20]. Furthermore, ROP18 was recently shown to phosphorylate at least one Thr residue on the host cell transcription factor ATF6β which leads to its degradation [21] and thus suppression of the host defence mechanisms in mice. The pseudokinase ROP5 (Chr.XII) was identified as another virulence factor [22,23].

South America and Asia are dominated by genetically different genotypes of *T. gondii*[24-26], some of which are highly mouse-virulent [27]. However, South American isolates appear also to be members of clonal lineages (e.g. BrI, BrII, BrIII and BrIV) that differ from the clonal lineages I, II and III [24,27,28]. There is evidence that *T. gondii* bearing predominantly non-type II alleles are often involved in ocular toxoplasmosis in humans [29-31]. *Toxoplasma gondii* that have alleles different from those observed in canonical clonal types I, II and III were shown to be associated with a number of severe toxoplasmosis cases in immunocompetent adults [32]. Experimental studies showed that non-canonical genotypes can develop when a cat ingests prey infected with *T. gondii* of more than one clonal type, followed by a sexual cross in the feline gut which can result in progeny representing a mixture of the two parental genotypes [18,33,34]. Sexual recombination is believed to be rare in nature while selfing of *T. gondii* seems to be the predominant route of reproduction and one of the reasons for its clonal population structure [16,35]. As a consequence, more than 95% of strains isolated from animals and humans in North America and Europe belong to the three main clonal lineages. However, it has been suggested that sexual recombination plays an important role in the diversification of *T. gondii* strains in nature [5,9,36], especially in South America [27] and Asia [37,38] where mixed genotypes were isolated by bioassay in mice from cats, chickens and sheep [27,39,40].

We previously reported the discovery of a single feline faecal sample containing *T. gondii* oocysts (namely TG-GER63) that showed combinations of type II and III alleles at several different loci [15]. In the present study, we isolated a number of genotypically different *T. gondii* clones from this individual faecal sample and observed that these clones showed major differences in virulence for BALB/c mice. Genetic markers presumed to be linked with virulence were applied on this set of *T. gondii* clones to examine whether the presence or absence of these markers were associated with virulent phenotypes.

## Methods

### Feline faecal sample

A feline faecal sample was collected from a cat by its owner in December 2008 and submitted to VetMed Labor GmbH, Ludwigsburg where it was examined by a conventional flotation method [15]. Floated material was transferred onto a slide and examined by light microscopy at a magnification of at least 200x. When oocysts with a diameter of about 9-14 μm were observed, the remaining sample was posted to the Friedrich-Loeffler-Institut, Wusterhausen, Germany. The sample was then examined by a sedimentation/flotation procedure as described [15]. Oocysts were counted using a Neubauer chamber and an aliquot of 1x10^4^ oocysts was used for DNA isolation. Oocysts were allowed to sporulate at room temperature for 96 hrs under aerated conditions and then stored in 1-2% (w/v) K_2_Cr_2_O_7_ at 4-8°C until use.

### DNA isolation

DNA was extracted from pellets of *in-vitro* grown tachyzoites using NucleoSpin® Tissue kit (Macherey-Nagel, Germany).

DNA was extracted from oocysts by chemical/mechanical destruction of the oocysts followed by phenol/chloroform extraction as described previously [15,41]. DNA was resolved in 100 μl sterile double-distilled water and stored at 4-8°C. Aliquots of 1 μl were used for PCR.

### Interferon-γ-knockout (GKO) and BALB/c mouse infections using oocysts

Sporulated oocysts kept in K_2_Cr_2_O_7_ solution were washed four times by centrifugation (1,100xg, 7 min, without brake) and resuspended in 15 ml water. The resulting pellet was finally resuspended in 1 ml water. Sporulated oocysts were counted using a Neubauer chamber. Two interferon-γ-knockout (GKO) -mice (C.129S7(B6)-Ifngtm1Ts/J, The Jackson Laboratory, USA) were infected by oral gavage containing 1x10^3^ sporulated oocysts in a volume of 0.2 ml. When the first clinical signs were detected, the mice were sacrificed and necropsied. Peritoneal fluid of one mouse was used to infect a VERO cell culture and to infect five BALB/c (BALB/cAnNCrl, Charles River, Germany) mice intraperitoneally [15]. Peritoneal fluid, heart and brain tissue samples of all infected mice were used to establish *T. gondii*-infected VERO cell cultures.

All animal experiments in this study were approved by the Ministerium fuer Landwirtschaft, Umweltschutz und Raumordnung of the German Federal State of Brandenburg.

### Virulence of *T. gondii* clones in BALB/c mice

Two to three months old BALB/c mice were used in all experiments. For intra-peritoneal infections, tachyzoites were grown and extracted from host cells by passage through a 27-gauge needle, washed two times in RPMI and quantified using a Neubauer chamber. Parasites were diluted in RPMI, and groups of five mice per dose inoculated intraperitoneally with 10^6^, 10^4^, 10^2^ or 10 tachyzoites of each clone (in 500 μl) using a 27-gauge needle. Each *in-vivo* experiment was carried out alongside a negative control group inoculated with RPMI only. Weight and mortality of the animals were recorded daily for 30 days after infection. Blood samples were taken at 0, 7, 14, 21, 28 and 30 days post-infection (d.p.i.). Mice were sacrificed at more than 20% weight loss or at the end of the experiment (30 d.p.i.). All animals were necropsied and their organs weighed. Virulence was categorised as high virulent (LD_50_ < 10^2^ tachyzoites), intermediate virulent (LD_50_ ≥ 10^2^ but <10^4^ tachyzoites) and low-virulent (LD_50_ ≥ 10^4^ tachyzoites) as described [27].

### Tissue culture and limiting dilution

All *T. gondii* isolates and reference strains (RH, Me49 and NED) were maintained in VERO cells and tachyzoites were isolated, purified and stored until used as described [15].

Limiting dilutions were carried out in 96-well-plates (Greiner, Germany). VERO cells were seeded at 1x10^6^ cells per well. Resulting monolayers were infected by adding 200 μl medium containing a calculated number of 0.1-0.5 parasites per well. When the first plaques were observed, clones were transferred into 25 cm^2^ tissue culture flasks (Greiner, Germany).

### PCR and PCR-RFLP

For all PCR reactions we used primers, dNTPs and Taq polymerase at a final concentration of 0.5 μM, 250 μM and 1 U/25 μl, respectively, with the buffer system supplied with the enzyme (all by Stratec Molecular, Germany).

DNA extracted from oocysts and tachyzoites was analysed by PCR using the common apicomplexan SSU-rDNA primers COC-1 and COC-2 [42], *T. gondii* specific primer pairs TOX4/TOX5 [43] and TOX5/Tox-8 [43,44].

All clones used in *in-vivo* mouse virulence studies were analysed by PCR. Absence or presence of the upstream region of ROP18 (UPS-ROP18) as potential indication of virulence was determined using primer pairs absenceROP18-F/absenceROP18-R and insertROP18-F/insertROP18-R as published [45]. Furthermore, a primer pair for CS3 [27] was used as another potential virulence marker.

Strain typing was performed using nine independent, unlinked genetic markers (nSAG2, SAG3, BTUB, GRA6, c22-8, c29-2, L358, PK1 and Apico) for PCR-RFLP as described [15,46]. PCR-amplified marker regions were digested with restriction endonucleases (Fermentas, Germany) and analysed as described [46]. In case of the *T. gondii* clones used for *in-vivo* studies, seven additional marker regions located at seven different chromosomes (AK16 [Chr.Ib], AK97 [Chr.III], AK22 [Chr.V], L53 [Chr.VI], AK53 [Chr.VIII] and L375 [Chr.IX]) were analysed by PCR-RFLP as described in the Toxoplasma Genome Mapping Database [47]. Three reference strains including RH, Me49 and NED were included in each PCR-RFLP run.

### Immunoblot

To detect antibodies directed against the immunodominant surface antigen TgSAG1 of *T. gondii*, previously established protocols were employed [48,49]. Mouse serum was diluted 1:10 in PBS-TG (PBS with 0.05% [v/v] Tween 20 (Sigma) and 2% [v/v] liquid fish gelatin [Serva, Germany]) and AffiniPure Rabbit Anti-Mouse IgG + IgM(H + L) (Jackson ImmunoResearch, USA) was diluted 1:250 in PBS-T (PBS with 0.05% [v/v] Tween 20 [Sigma]). Test results were regarded as positive when the TgSAG1 band at 30 kDa was detected.

## Results

### Isolation of *T. gondii* clones and genotyping

To identify different individual genotypes in the oocyst field sample, two GKO mice were orally infected with 1x10^4^ sporulated oocysts from TG-GER63. Both mice showed first clinical signs 8 d.p.i. and were sacrificed. *Toxoplasma gondii* tachyzoites were isolated from these mice and cultured on VERO cells (K119/1 and K119/2). Genotyping of the two isolates revealed a mixture of type II and III alleles at several individual loci (Table 1) similar but not identical to the pattern observed in the original isolate TG-GER63 (Table 1). Tachyzoites harvested from the K119/1 cell culture were used to infect five BALB/c mice (B136/1, B136/2, B136/3, B136/4 and B136/5) which died 20, 22, 23, 21 or 21 d.p.i., respectively. *Toxoplasma gondii*-infected VERO cell cultures, one from each mouse, were established using peritoneal washings. All *T. gondii* tachyzoites isolated from infected GKO and BALB/c mice showed a mixture of type II and III alleles at different loci (Table 1).

**Table 1:** Multilocus genotyping of *Toxoplasma gondii* isolates and clones originating from a single oocyst sample by PCR-Restriction Fragment Length Polymorphism (PCR-RFLP) analysis

**Designation**	**Inoculum**	PCR-RFLP genotype - genetic marker (location*)								
		**nSAG2 (Chr.VIII)**	**SAG3 (Chr.XII)**	**BTUB (Chr.IX)**	**GRA6 (Chr.X)**	**c22-8 (Chr.Ib)**	**c29-2 (Chr.III)**	**L358 (Chr.V)**	**PK1 (Chr.VI)**	**Apico (Plastid)**
RH		I	I	I	I	I	I	I	I	I
Me49		II	II	II	II	II	II	II	II	II
NED		III	III	III	III	III	III	III	III	III
TG-GER63^a^		I or III + II	II	II	III	II + III	II + III	III	II + III	III
K119/1^b^	Oocysts (TG-GER63)	III	II	III	II + III	II + III	II + III	II + III	II	III
K119/2	Oocysts (TG-GER63)	III	II	III	III	II + III	III	III	III	III
B136/1^c^	Tachyzoites (K119/1)	II + III	III	III	II + III	II + III	III	II + III	II + III	III
B136/2	Tachyzoites (K119/1)	II + III	III	III	II + III	III	III	II + III	II + III	III
B136/3	Tachyzoites (K119/1)	II + III	III	III	II + III	III	III	II + III	II + III	III
B136/4	Tachyzoites (K119/1)	II + III	III	II + III	II + III	III	III	II + III	II + III	III
B136/5	Tachyzoites (K119/1)	II + III	III	III	II + III	II + III	III	II + III	II + III	III

Two interferon-γ-knockout (GKO) mice (K119/1 and K119/2) were orally inoculated with sporulated oocysts of TG-GER63^a^. Tachyzoites isolated from the peritoneal fluid and brain tissue of K119/1 were used to infect five BALB/c mice (B136/1-5) intra-peritoneally and to establish *in-vitro* tissue cultures of these parasites in VERO cells. Three reference strains including RH, Me49 and NED were included in each PCR-RFLP run.

^a^ as described by [15].

^b^ K, *T. gondii* cell-culture clone obtained from infected GKO mouse.

^c^ B, *T. gondii* cell-culture clone obtained from infected BALB/c mouse.

* Chr., chromosome.

Several rounds of limiting dilution resulted in the isolation of 45 *T. gondii* clones from mixed *T. gondii* VERO cell cultures. Most clones showed a combination of type II and III alleles at several different loci. Clones displaying the same allele combinations over all examined loci were thus grouped accordingly (Table 2). In total, five genotypically different *T. gondii* clone groups, based on their PCR-RFLP allele pattern, could be identified. *Toxoplasma gondii* clones within four groups showed a non-canonical allele pattern that have never been described in any intermediate or definitive host species before (groups 2-5). Group 1 contained sixteen clones displaying type III alleles at all loci. All twenty-two clones of group 2 showed alleles of type III at all loci except for a type II allele at PK1 (Chr.VI). Clones within group 3 displayed type II alleles at the BTUB (Chr.IX) and c29-2 (Chr.III) loci with all remaining loci sharing type III alleles. All clones in group 4 displayed type III alleles except for type II alleles at nSAG2 (Chr.VIII), c22-8 (Chr.Ib), L358 (Chr.V) and PK1. Group 5 contained one clone which is almost identical with members of group 4 except for a type I allele at the Apico locus.

**Table 2:** Multilocus genotyping of *Toxoplasma gondii* clones isolated from *in-vitro* cell cultures of *T. gondii* by limiting dilution

**Designation**	**Origin (number of clones)**	PCR-RFLP genotype - genetic marker (location*)								
		**nSAG2 (Chr.VIII)**	**SAG3 (Chr.XII)**	**BTUB (Chr.IX)**	**GRA6 (Chr.X)**	**c22-8 (Chr.Ib)**	**c29-2 (Chr.III)**	**L358 (Chr.V)**	**PK1 (Chr.VI)**	**Apico (Plastid)**
Group 1 (16 clones)	K119/2 ^a^ (10), B136/4 ^b^ (4),K119/1 (1), B136/5 (1)	III	III	III	III	III	III	III	III	III
Group 2 (22 clones)	B136/2 (13), K119/1 (4), K119/2 (4), B136/4 (1)	III	III	III	III	III	III	III	II	III
Group 3 (2 clones)	K119/2 (2)	III	III	II	III	III	II	III	III	III
Group 4 (4 clones)	B136/1 (3), B136/5 (1)	II	III	III	III	II	III	II	II	III
Group 5 (1 clone)	B136/4 (1)	II	III	III	III	II	III	II	II	I

Typing was performed by PCR-Restriction Fragment Length Polymorphism (PCR-RFLP) analysis. *T. gondii* clones were grouped according to their allele pattern.

^a^ K, *T. gondii* cell culture clone obtained from infected GKO mouse.

^b^ B, *T. gondii* cell culture clone obtained from infected BALB/c mouse.

* Chr., chromosome.

While type II alleles were initially observed at the SAG3 locus (Chr.XII) in cell cultures infected with *T. gondii* of K119/1 and K119/2, none of the subsequently isolated clones displayed a type II allele at that locus. Mixed cell cultures isolated from K119/1 always showed a mixture of type II and III alleles at the GRA6 locus (Chr.X) but no type II allele was observed at this particular locus in any resulting clone.

To investigate whether *T. gondii* clones of different allelic patterns were the result of a recombination event or of a re-assortment of chromosomes in the definitive host, additional chromosomal markers were studied in one or two clones from each clone group (Table 3). No allelic type differences were observed between the genetic markers used for genotyping and the additional chromosomal markers in *T. gondii* clones of clone groups 1 (K119/2 2-H8, K119/2 2 F3-A3) and 2 (K119/2 A7, B136/2 C12). Interestingly, *T. gondii* clones of clone group 3 (K119/2 2-C10, K119/2 G11-C4) showed alleles of different types on Chr.V, while type III alleles were observed on this chromosome using the genetic marker L358, a type II allele was detected using the genetic marker AK22. Clones in clone group 4 (B136/5 G12, B136/1 B6-H6) and 5 (B136/1 A3-F5) had a type III allele at locus c29-2, but a type II allele at locus AK97, both of which are located on Chr.III. Furthermore, a type II allele was observed at the nSAG2 locus, but a type III allele at the L375 locus, both of which are located on Chr.VIII.

**Table 3:** Multilocus genotyping applying additional chromosomal markers of *Toxoplasma gondii* clones to selected clones used in virulence study

Chr.*	**Marker ID**	***T. gondii*clones**								
		**Group 1**		**Group 2**		**Group 3**^**§**^		**Group 4**^**§**^		**Group 5**^**§**^
		**K119/2 2-H8**	**K119/2 2-F3-A3**	**K119/2 A7**	**B136/2 C12**	**K119/2 2-C10**	**K119/2 G11-C4**	**B136/5 G12**	**B136/1 B6-H6**	**B136/1 A3-F5**
Ib	c22-8^a^	III		III		III		II		II
	AK16^b^									
III^§^	c29-2^a^	III		III		II		III		III
	AK97^b^							II		II
V^§^	L358^a^	III		III		III		II		II
	AK22^b^					II				
VI	PK1^a^	III		II		III		II		II
	L53^b^									
VIII^§^	nSAG2^a^	III		III		III		II		II
	AK53^b^							III		III
IX	BTUB^a^	III		III		II		III		III
	L375^b^									
X	GRA6^a^	III		III		III		III		III
	AK69^b^									
XII	SAG3^a^	III		III		III		III		III
plastid	Apico^a^	III		III		III		III		I

Typing was performed by PCR-Restriction Fragment Length Polymorphism (PCR-RFLP) analysis. *T. gondii* clones were grouped according to their allele pattern.

^a^ Su et al. (2006) [46].

^b^ Toxoplasma Genome Mapping Database [47].

* Chr., chromosome.

^§^ evidence for sexual recombination.

### Virulence of *T. gondii* clones

Up to two clones from each clone group were randomly chosen to determine their *in-vivo* virulence in BALB/c mice using different tachyzoite doses of each clone as indicated in Table 4. All mice used in these experiments were tested for seroconversion by immunoblot analysis upon death or at the end of the experiment to confirm infection (30 d.p.i.). All mice inoculated with *T. gondii* seroconverted except mice that died before 10 d.p.i. and six mice inoculated with only 10 parasites (three from group B136/1 B6-H6 and three from group B136/1 A3-F5).

**Table 4:** Mouse virulence of *T. gondii* clones

**Clone**	**Clone**	**UPS-ROP18**^**a**^	**CS3**^**b**^	**% Mortality in mice (No. dead/No. infected)**					**LD**_**50**_	**Virulence**
	**group**			**10**^**6c**^	**10**^**4c**^	**10**^**2c**^	**10**^**c**^	**0**^**c**^		
K119/2 2-H8^d^	1	III	III	20 (1/5)	*n.d*.^e^	*n.d.*	*n.d.*	0 (0/5)	>10^6^	low
K119/2 2 F3-A3	1	III	III	20 (1/5)	*n.d.*	*n.d.*	*n.d.*	0 (0/5)		
K119/2 A7	2	III	III	100 (5/5)	100 (5/5)	0 (0/5)	*n.d.*	0 (0/5)	>10^2^, <10^4^	intermediate
B 136/2 C12^f^	2	III	III	100 (5/5)	100 (5/5)	100 (5/5)	20 (1/5)	0 (0/5)	>10, <10^2^	high
K119/2 2-C10	3	I / II	II	0 (0/5)	*n.d.*	*n.d.*	*n.d.*	0 (0/5)	>10^6^	low
K119/2 G11-C4	3	I / II	II	0 (0/5)	*n.d.*	*n.d.*	*n.d.*	0 (0/5)		
B136/5 G12	4	I / II	II	100 (5/5)	100 (5/5)	60 (3/5)	20 (1/5)	0 (0/5)	>10, <10^2^	high
B136/1 B6-H6	4	I / II	II	100 (5/5)	100 (5/5)	100 (5/5)	100 (2/2)^g^	0 (0/5)	<10	
B136/1 A3-F5	5	I / II	II	*n.d.*	100 (5/5)	60 (3/5)	50 (1/2)^g^	0 (0/5)	10	high

Virulence of *T. gondii* clones for BALB/c mice was predicted using virulence markers for the upstream region of ROP18 (UPS-ROP18) and CS3. To determine the *in-vivo* mouse virulence of the clones, five BALB/c mice were each infected with 10^6^, 10^4^, 10^2^, 10 and 0 tachyzoites i.p. per clone.

^a^ virulence marker UPS-ROP18 published by [45].

^b^ virulence marker CS3 published by [27].

^c^ number of *T. gondii* tachyzoites injected i.p. per mouse.

^d^ K, *T. gondii* cell culture clone obtained from infected GKO mouse.

^e^*n.d.* not done.

^f^ B, *T. gondii* cell culture clone obtained from infected BALB/c mouse.

^g^ only two of five infected mice were seropositive in the immunoblot.

Representatives of clone groups 1 and 3 were of low virulence in mice. Infecting mice with 10^6^ tachyzoites i.p. resulted in few mouse deaths (2 out of 20). Representatives of clone group 2 showed an intermediate (LD_50_ ≥ 10^2^ but <10^4^ tachyzoites) or high (LD_50_ <10^2^ tachyzoites) virulence phenotype in mice. All representatives of clone group 4 and 5 were highly virulent (LD_50_ < 10^2^ tachyzoites) in mice.

**Figure 1:**
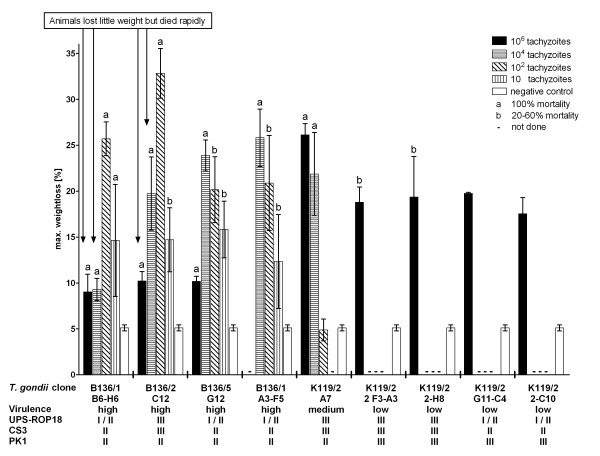
**Experimental infection of BALB/c mice.** Five animals per group were infected with 10^6^, 10^4^, 10^2^ or 10 *T. gondii* tachyzoites i.p. each. Means of maximum weight losses, genetic marker PK1 and virulence markers UPS-ROP18 [45] and CS3 [27] are show.

All mice lost weight after infection with *T. gondii*, irrespective of the genotype or the infection dose. Weight loss and dose of infection were positively correlated, except for high-dose challenges with virulent clones which killed the mice rapidly. In contrast, mice infected with low-virulent *T. gondii* clones and negative control animals only lost up to 5% of weight. Infection with high doses (10^6^ and 10^4^ parasites) of high-virulent clones (group 2 and 4) led to weight losses of only up to 10%, but the animals died within 2-3 days after the onset of disease. However, mice infected with lower doses of high-virulent clones had lost more than 15% of weight when they died, in some cases even more than 30%. Some animals showed a rapid decrease from less than 20% to 30% weight loss within 24 hours (Figure 1).

When the route of *in-vitro* isolation was analysed, it became apparent that all highly mouse-virulent clones had been passaged through BALB/c mice prior to *in-vitro* isolation, while all clones isolated after a GKO mouse passage were of low or intermediate virulence.

### Molecular virulence markers

Predictions using the virulence marker CS3 corresponded perfectly with the predictions of UPS-ROP18 (Table 4). UPS-ROP18 was absent (type I/II) in all representatives of clone groups 4 and 5 (LD_50_ < 10^2^ tachyzoites) which correctly predicted their high virulence for mice. UPS-ROP18 was present (type III) in representatives of clone group 1 (LD_50_ > 10^6^ tachyzoites) which correctly predicted their low virulence for mice. However, UPS-ROP18 was absent in representatives of clone group 3 (LD_50_ > 10^6^ tachyzoites), thus predicting high virulence for mice, which is in contrast to their low virulence for BALB/c mice. Representatives of clone group 2 showed intermediate (LD_50_ ≥ 10^2^ but <10^4^ tachyzoites) and high (LD_50_ > 10 tachyzoites but <10^2^ tachyzoites) virulence for BALB/c mice despite the presence of UPS-ROP18 and CS3 type III, which have been proposed as a predictor of low mouse virulence [45]. The clones described here showed a positive correlation between virulence and the PK1 allele analysed by PCR-RFLP. All mouse-virulent clones had a type II allele at the PK1 locus, whereas non-virulent clones had a type III allele present at this locus (Figure 1).

## Discussion

To our knowledge, this is a first study demonstrating evidence for sexual recombination occurring in a felid under naturally conditions. A large number of genotypically different *T. gondii* clones with differences in mouse virulence were recovered from oocysts present in a single feline faecal sample isolated from a naturally infected cat. PCR-RFLP analysis at nine genetic loci revealed *T. gondii* clones of five genotypes. Most clones displayed various combinations of type II and type III alleles at the analysed loci while some displayed only type III alleles. Although these clones were genotypically identical to the clonal type III, we cannot exclude that typing with additional markers may lead to the detection of alleles other than those of type III in at least some of these clones. The same may also apply to any of the clones within each group of the non-canonical types described in this study. Different mouse virulence of *T. gondii* clones with an identical allele pattern in group 2 support this hypothesis.

In Europe, *T. gondii* of clonal type II prevails [45], but in Spain, Portugal, Poland and Germany genotypes different from clonal type II were previously described in chickens [38], pigs [12] and pigeons [14]. *Toxoplasma gondii* of type II and III were recently found in cats from Germany [15]. Therefore, sexual recombination events caused by the almost simultaneous ingestion of *T. gondii* of different types by a single feline definitive host seem to be possible, but rare, in Europe. Sexual crosses may lead to a large number of new recombinant *T. gondii* genotypes shed as oocysts. Recent studies, describing recombinant and mixed *T. gondii* types in intermediate hosts from European countries [38,50] support this hypothesis. Recombination events in cats were so far only shown experimentally [17,18,34]. An analysis of *T. gondii* isolates from a waterborne toxoplasmosis outbreak in Brazil found several *T. gondii* genotypes which may have been the result of a sexual recombination event [51].

The *T. gondii* oocysts used in this study where isolated directly from a single feline faecal sample. This suggests that a sexual cross has occurred in this felid under naturally conditions. For *T. gondii* clones of group 1 and 2 the oocysts may be the result of re-assortment of chromosomes in the cat the faecal sample derived from. However, since alleles of different types where observed at a single chromosome in *T. gondii* clones of group 3, 4 and 5, these clones seem to be the result of a sexual recombination, rather than chromosome sorting. Sexual recombination as well as chromosome sorting could have resulted from ingesting one or several intermediate hosts infected with genetically different *T. gondii* by the feline host. Of course, a single cat could have also taken up type III and different non-canonical *T. gondii*, thus shedding such genotypes with its faeces. However, considering the low prevalence of non-canonical types in Germany [15,52] this hypothesis seems less likely. Furthermore, the fact that we observed only type II and type III alleles in the chromosomal markers might suggest that type II and type III *T. gondii* were involved as parental strains as shown in previous experimental studies [17].

The detection of clones with large differences in mouse virulence in a single feline faecal sample is more important than the observation of a large variety of genotypes in this oocyst isolate. While both, type II and type III alleles were observed at different loci of the original isolate TG-GER63, the isolation procedure via GKO mice, BALB/c mice and cell culture, including limiting dilution, obviously caused a bias towards type III alleles in the resulting clones. Some alleles, like the type II allele at SAG3 and the type III allele at GRA6, were detected in the original oocyst sample TG-GER63, but not in any of the resulting clones. Moreover, the isolation procedure also may have had an effect on the virulence phenotypes of the resulting clones. All low- or intermediate-virulent clones were derived from GKO mice. In contrast, all highly virulent clones were obtained after passage in BALB/c mice. This suggests that exposure of the parasites to an immune system which is not impaired, unlike that of GKO mice, may have favoured parasites with higher mouse virulence, for example subpopulations which can replicate faster under the pressure of the host immune system. This may also explain why some clones arising after a sexual crossing event expand better in a given host system while others disappear. It is tempting to speculate that the intermediate host species infected by the arising clones has a significant influence on this selection processes, e.g. via the immune system or other factors influencing parasite replication or persistence.

Infection of domestic or wild animal species (including rodents or birds) may lead to the expansion of completely different *T. gondii* genotypes [53]. In countries where domestic felids are less abundant, wild felids such as jaguars in French Guiana may serve as definitive hosts [54]. As a consequence, domestic and sylvatic cycles may exist in parallel or even overlap. Observations of mixed genotypes in indigenous prey like free-range chickens in South America [25,26,55], deer, cougars, raccoons and skunks in North America [56] indicate the importance of the sylvatic cycle as a driving force for genetic diversity in *T. gondii*. It was suggested that new *T. gondii* strains originating from different prey in the sylvatic cycle capable of clonal expansion may sweep the domestic cycle [53].

*Toxoplasma gondii* possesses a number of virulence genes that show an altered biological potential in recombinant strains. It has been shown that alleles of the ROP18 and ROP16 genes possess virulence-potentiating properties [17,18]. There are only three major lineages of ROP18 (ROP18I*, ROP18II* and ROP18III*) evident world-wide, corresponding to the three clonal types I, II and III, respectively. The vast majority of South American isolates share alleles of the ROP18I* lineage which is associated with high virulence in mice. ROP18I* and ROP18II* lack the presence of an upstream region [9] whereas ROP18III* is always flanked by this region. Almost all isolates that possessed the ROP18III* allele were avirulent in mice. We found, however, that the virulence of the clones we had isolated did not always correspond to the absence or presence of UPS-ROP18. In fact, four clones (K119/2 2-C10, K119/2 G11-C4, B136/2 C12 and K119/2 A7) were not in agreement with the virulence prediction based on the presence or absence of UPS-ROP18. Since two isolates (CASTELLS and P89) had also failed to conform to the UPS-ROP18 virulence predictions [45], there must be additional factors influencing the virulence of *T. gondii* isolates. Surprisingly, the prediction of mouse virulence using the presence or absence of UPS-ROP18 and CS3 corresponded perfectly, although the regions coding for these genes are approximately 133 kbp apart in the *T. gondii* (strain Me49) genome [57]. Another virulence factor that could be involved here, namely ROP5, has recently been described [22,23,58]. Analysis of recombinant progeny derived from the genetic cross between type I and type II that showed marked allelic difference between type I and type II correlating with different levels of mouse virulence. Such allelic differences contain SNPs confined to the ATP-binding pocket of ROP5 [23].

Interestingly, the marker PK1, used for genotyping purposes, matched the virulence phenotypes of all the isolates used in our study. All virulent clones possessed the type II allele of PK1, while the type III allele was observed in avirulent clones. Sequencing a natural type II/III *T. gondii* recombinant (TgCkUg2) obtained from chickens in Uganda revealed that it arose via chromosome sorting and not by interchromosomal recombination, possibly through a single recombination event [40]. The correlation of the virulence phenotype with PK1 does therefore not necessarily mean that the potential virulence associated gene is located close to this locus, but rather that it is located on the same chromosome as PK1, i.e. Chr.VI [18]. However, this observation might be coincidental, as a correlation between PK1 and mouse virulence has not been shown in any of the highly diverse isolates from Brazil [27]. This may also suggest that a particular virulence phenotype does not necessarily require the inheritance of specific alleles. Reshuffling of existing alleles at a few loci seems to be sufficient to produce new *T. gondii* clonal types with different biological traits [29,36,53]. Nevertheless, future studies should focus on the high-resolution characterisation using next generation sequencing of the clones described in this study. Together with sequencing data of atypical and other non-canonical *T. gondii* isolates whose virulence is known, new virulence determining factors and more reliable virulence markers could be developed in the future.

This study and our previous work [15,41] clearly showed that - although clonal type II *T. gondii* prevail in our region - new clonal types can arise under these natural conditions as a result of a sexual cross. Emerging new clonal types may show different levels of virulence in intermediate hosts. In the present study this was only shown for BALB/c mice. However, it is not unlikely that new clonal types with increased virulence in humans or other intermediate hosts may arise as a result of sexual recombination of *T. gondii* in felids. Currently, no data exist on the distribution of *T. gondii* genotypes in human toxoplasmosis cases in Germany that could be correlated with the current genotypes found in cats.

## Competing interests

The authors declare that they have no competing interests.

## Authors’ contributions

The original faecal sample containing *T. gondii* oocysts was identified and supplied by MGV and NP. DCH and GS initiated and conducted the *in-vivo* experiments, coordinated the experimental design, analysis and interpretation of data and wrote the manuscript. DCH isolated oocysts from the original TG-GER63 sample. DCH, AB and AM conducted *in-vitro* experiments and isolated individual *T. gondii* clones. DCH and AM carried out genotyping of *T. gondii*. FJC assisted with the study design and writing of the manuscript. All authors have read and approved the final manuscript.
